# Severe head injury in children: intensive care unit activity and mortality in England and Wales

**DOI:** 10.3109/02688697.2010.538770

**Published:** 2010-11-17

**Authors:** ROBERT C TASKER, THOMAS J FLEMING, AMBER ER YOUNG, KEVIN P MORRIS, ROGER C PARSLOW

**Affiliations:** 1 Department of Paediatrics, Cambridge University Clinical School, Addenbrooke's Hospital, Cambridge, UK; 2 Data Management Group, Centre for Epidemiology and Biostatistics, University of Leeds, Leeds, UK; 3 Department of Anaesthesia, Frenchay Hospital, Bristol, UK; 4 Paediatric Intensive Care Unit, Birmingham Children's Hospital, Birmingham, UK; 5 Paediatric Epidemiology Group, Centre for Epidemiology and Biostatistics, University of Leeds, Leeds, UK

**Keywords:** Head injury, severity of illness, neurosurgical intensive care, risk-adjusted mortality, severity of illness

## Abstract

**Objective:**

To explore the relationship between volume of paediatric intensive care unit (PICU) head injury (HI) admissions, specialist paediatric neurosurgical PICU practice, and mortality in England and Wales.

**Methods:**

Analysis of HI cases (age 516 years) from the Paediatric Intensive Care Audit Network national cohort of sequential PICU admissions in 27 units in England and Wales, in the 5 years 2004-2008. Risk-adjusted mortality using the Paediatric Index of Mortality (PIM) model was compared between PICUs aggregated into quartile groups, first to fourth based on descending number of HI admissions/year: highest volume, medium-higher volume, medium-lower volume, and lowest volume. The effect of category of PICU interventions - observation only, mechanical ventilation (MV) only, and intracranial pressure (ICP) monitoring - on outcome was also examined. Observations were reported in relation to specialist paediatric neurosurgical PICU practice.

**Results:**

There were 2575 admissions following acute HI (4.4% of non-cardiac surgery PICU admissions in England and Wales). PICU mortality was 9.3%. Units in the fourth-quartile (lowest volume) group did not have significant specialist paediatric neurosurgical activity on the PICU; the other groups did. Overall, there was no effect of HI admissions by individual PICU on risk-adjusted mortality. However, there were significant effects for both intensive care intervention category (*p*<0.001) and HI admissions by grouping (*p*<0.005). Funnel plots and control charts using the PIM model showed a hierarchy in increasing performance from lowest volume (group IV), to medium-higher volume (group II), to highest volume (group I), to medium-lower volume (group III) sectors of the health care system.

**Conclusions:**

The health care system in England and Wales for critically ill HI children requiring PICU admission performs as expected in relation to the PIM model. However, the lowest-volume sector, comprising 14 PICUs with little or no paediatric neurosurgical activity on the unit, exhibits worse than expected outcome, particularly in those undergoing ICP monitoring. The best outcomes are seen in units in the mid-volume sector. These data do not support the hypothesis that there is a simple relationship between PICU volume and performance.

## Introduction

In the UK during 2001 to 2003 the incidence of head injury (HI) in childhood necessitating intensive care management was 5.6 per 100,000 paediatric population per year.^1^ Emergency care for these children is organised such that urgent supportive management is initiated locally and subsequent intensive care of intracranial complications is undertaken in regional^2^—^4^

centres.

Two health care system issues arise from regional centralisation of services: how does the provision of emergency practice contend with the geographical problem of patient access, particularly if timeliness (e.g. neurosurgery within four hours) is a key requirement?; and, given the geography of population density, does volume of paediatric intensive care unit (PICU) HI practice have an impact on mortality? We know from recent work that the system of access to emergency neurosurgery in severely head injured children in England and Wales does not achieve surgical evacuation of a haematoma in a timely manner.^5^,^6^ This problem is being addressed by the UK Department of Health.^7^,^8^ In regard to regional differences in practice, there is significant variation in PICU management of intracranial pressure (ICP) complicating severe HI.^9^ The lack of standardisation is not explained, but may relate to differences in paediatric neurosurgical caseload in the PICU, and what this activity implies in regard to experience and collective team expertise. In this report we have used the Paediatric Index of Mortality (PIM) risk adjustment model to explore the relationship between volume of PICU HI practice and mortality in England and Wales.

## Methods

The National Information Governance Board (formerly the Patient Information Advisory Group, see http://www.nigb.nhs.uk/ecc/reg/regoutput.xls) has approved of the collection of the personally identifiable data used in this report and ethics approval was granted by the Trent Medical Research Ethics Committee (reference 05/MRE04/17).

### Dataset

Over the period 1 January 2004 to 31 December 2008 there were 58,045 episodes of non-cardiac surgery paediatric intensive care carried out in 29 PICUs in England and Wales. We have examined the standard dataset of demographic and clinical information and PICU mortality on these episodes that is collected by PICU staff using bespoke software provided by the Paediatric Intensive Care Audit Network (PICANet). Since 2002 PICANet has managed this system of audit centrally and performed regular unit and staff training on data definitions and data collection. The organisation also carries out regular local and central validation checks in order to ensure consistent data quality between units.^10^,^11^

### Selection of cases

Episodes of paediatric intensive care for acute management of HI in under 16 year olds were extracted from the PICANet dataset by searching all recorded levels of diagnosis and procedures for terms related to HI and head trauma. This process involved first searching all neurological Clinical Terms 3 (the Read codes) used in the PICANet dataset. We identified 233 terms that related to any form of HI. Each episode with these codes was then reviewed to ensure that the reason for admission was acute HI. Out of all 233 terms there were, in most common usage, 68 related to diagnosis and 8 to operative procedures.

In order to explore any relationship between total PICU caseload, type of paediatric neurosurgical PICU caseload, and HI outcome we collected data on total admissions as well as caseload that fell into the following categories: hydrocephalus (e.g. shunt and other procedures); central nervous system (CNS) tumours; spinal surgery not scoliosis; neuro-vascular conditions not related to HI; and, other specialist neurosurgical work (e.g. cerebral abscess, craniofacial surgery, epilepsy surgery, and interventions for CNS malformations).

### Data

All data were anonymised with blinding of the PICU where the episode of care occurred. Data extracted from the PICANet dataset included length-of-stay in days (calculated from the dates and times of admission and discharge), and the child's age, sex and outcome (alive or died) at PICU discharge. The HI admission rate per year for each PICU was calculated by dividing the sum of the episodes for that unit by the number of years of PICANet data collection in that unit.

We also extracted information about PIM ‘risk of mortality’ for each episode of care, along with the component information that makes this calculation possible.^12^ These data are collected between first contact with a PICU doctor and up to 1 hour after admission. The acute physiology used in the PIM logit calculation are systolic blood pressure, base excess in arterial or capillary blood, and the ratio of arterial partial pressure of oxygen to fractional inspired oxygen level. Glasgow Coma Scale (GCS) score is not used in the PIM instrument. The only factor related to brain function that is used in PIM (and also collected by PICANet since 2002) is whether or not pupillary reaction is abnormal. An abnormal response is when *both* pupils are fixed and more than 3 mm dilated on exposure to ‘strong direct light'. (The response should only be recorded as fixed when it is not due to ‘toxin, drugs, a local injury to the eye, or chronically altered from a previous disease').^13^

Our dataset (2004-2008) spans the period before and after the introduction of the recent recalibration of PIM (i.e. PIM2^14^) in June 2005.^15^ We have therefore undertaken all analyses using both versions of the model. Since the PIM2 recalibration incorporates additional non-physiological components to the score we have had to assume that these variables were normal in the earlier data collection period.

Last, as a marker of increasing HI severity that evolves over the course of illness, we extracted information about three interventions that may have been carried out at some time during the admission: use of mechanical ventilation (MV) via an endo-tracheal tube, laryngeal mask or tracheostomy; use of a ventricular drain or other ICP monitoring device; and use of an intravenous vasoactive drug such as dopamine, epinephrine and norepinephrine, etc.

### Statistical analysis

All statistical analyses were performed using either JMP 7.0 Statistical Discovery Software (SAS Institute Inc., Cary, NC, USA) or Microsoft Excel. Continuous variables were summarised by mean and 95 percent confidence interval (95% CI), or median and interquartile range (IQR) for skewed variables. Comparisons between groups were made using parametric or non-parametric tests. Statistical significance was set with *p*< 0.05.

#### Paediatric risk prediction in head injuries admissions

We used standard analyses to check the suitability of the PIM risk model in the HI population including measures of discrimination (i.e. the ability of the model to distinguish survivors from non-survivors),^16^,^17^ calibration (i.e. the accuracy of the estimated probability of survival),^18^,^19^ and overall fit.^20^,^21^ The suitability of the PIM risk model in the HI population was based on whether these descriptive parameters were similar to the values described as ‘good’ by the *Intensive Care National Audit and Research Centre* in their comparison of risk prediction models for adult HI.^21^

After the above evaluation, we used the PIM log odds of mortality in a logistic regression model with death as the outcome and assessed for risk-adjusted effects in the model, such as individual PICU volume, intensive care intervention for HI, and PICU grouping by caseload. Standardised mortality ratios (SMRs) were calculated by dividing the observed mortality by an expected mortality calculated from the PIM model. This value, with its confidence interval, is presented in instances where there were more than 20 deaths observed.

#### Funnel plots and risk-adjusted mortality

We used funnel plots to display risk-adjusted death rate and summarise performance of the PICU HI care system on a case series basis. We considered four quartile-sectors of the system based on size of PICU HI practice (first to fourth in descending order). We also considered individual PICUs within each quartile-sector. The PIM2 model was used in these assessments because it provides the most recent reference for mean predicted percentage death rate for 2004-2008.^15^ Upper and lower control limits were calculated at 3σ (calculated similarly to 99.8% confidence intervals, although control limits are prediction limits not precision limits) using an exact binomial method.^22^,^23^ We also calculated an upper warning limit (calculated similarly to 95% confidence intervals) at 2σ above the predicted mean. The lower 2σ limit was taken to indicate examples of possible best practice. Risk-adjustment of the data for each quartile in the PICU system was calculated as (observed outcome/expected outcome) × series-wide outcome.^23^,^24^ Risk-adjustment of the data for individual PICUs within a quartile-sector was calculated as (observed outcome/expected outcome) × outcome specific to the sector in which the PICU is a part.

#### Risk-adjusted control charting

Case series performance in each quartile was assessed by risk-adjusted control charts.^25^ The control chart is constructed using sequential probability ratio tests to test the hypothesis, H_A_, that the odds of death in the quartile-sector has doubled relative to the population used to calibrate PIM2; against the alternative hypothesis, H_0_, that the odds of death in the quartile-sector has not doubled.^26^ Results are plotted with the *x*-axis representing each patient in sequence and the *y*-axis representing the cumulative log likelihood ratio for a doubling of the odds of death. Control limits of 4.6 and -4.6 on the *y*-axis represent critical decision thresholds at which it may be inferred that the accumulated data support H_A_ or H_0_, respectively with Type I and Type II errors of 1% (α = 0.01, β = 0.01). The limits of 2.9 and -2.9 support H_A_ or H_0_ with Type I and Type II errors of 5% (α = 0.05, β = 0.05). In order to avoid the influence of accumulated credit (and therefore be able to detect earlier in the series better- or worse-than-expected outcomes) we have plotted a half-cumulative-sum-risk-adjusted chart.^25^ There are two lines in the chart. The upper line tests for doubling of the odds of death. The line is reset to zero in preference to allowing negative values, or if the upper threshold is crossed (h = 4.6). The lower line tests for halving of the odds of death. The line is reset to zero in preference to allowing positive values, or if the lower threshold is crossed (*h* = -4.6).

## Results

In the 5 years, 2004-2008, there were 2575 admissions following acute HI in the PICANet dataset. This total represents 4.4% of all non-cardiac surgical intensive care admissions to PICUs in England and Wales. These episodes of intensive care were managed in 27 PICUs and the patients were aged 8.5 (3.1-12.8) years, median (IQR). Two-thirds were boys, 4.9% had complicating seizures or status epilepticus, and 2.1% suffered a cardiac arrest or event requiring cardiopulmonary resuscitation. Injuries involving other parts of the body occurred in 9.4% of episodes. These included: upper or lower limb, 6%; thorax or lung, 1.6%; abdomen or multiple injuries 1.8%. Overall 239 children died (mortality 9.3%).

**Figure 1:**
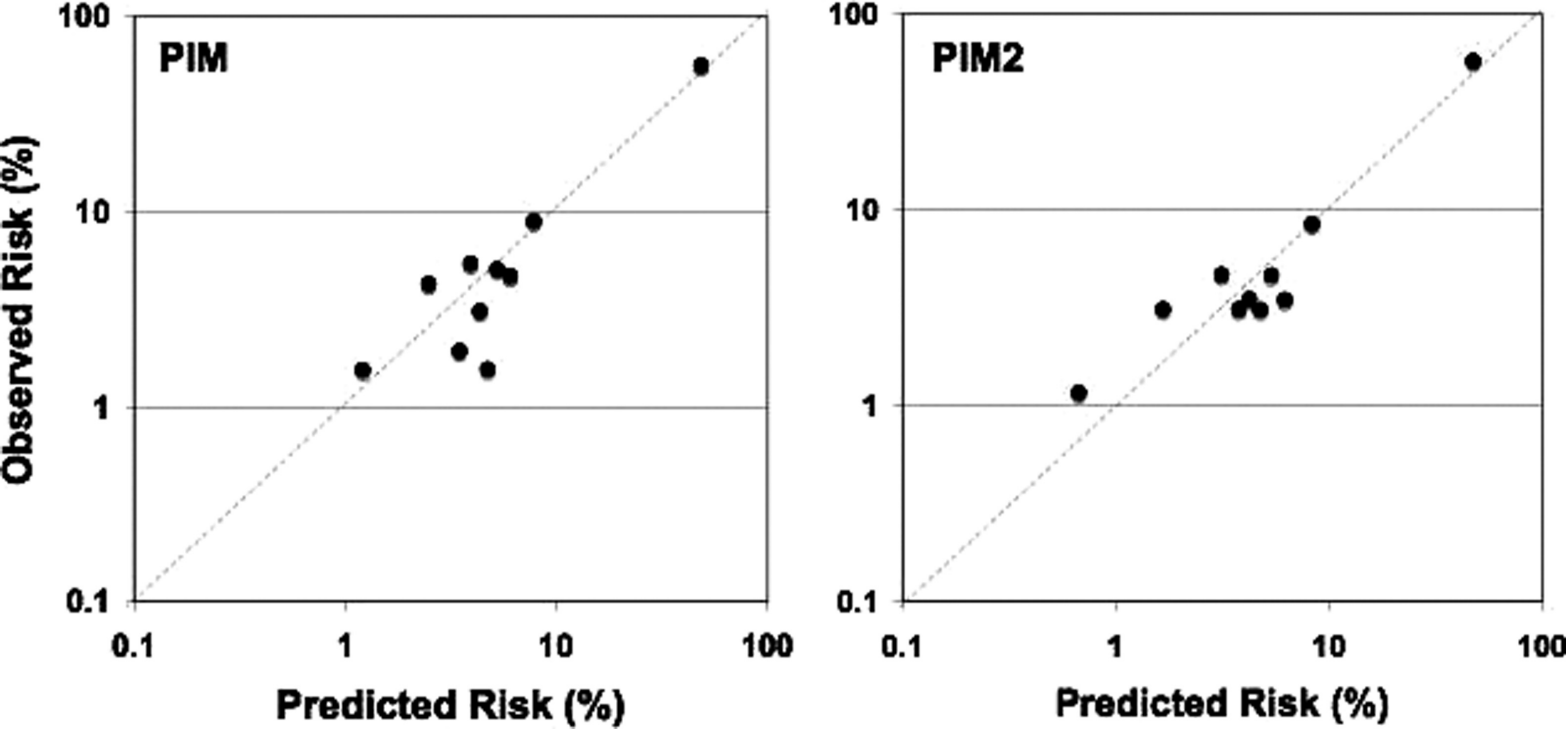
Calibration plots for PIM. The two plots show the observed risk by expected risk in deciles of expected risk using a log scale for the two versions of PIM.

The calibration of the PIM model in child HI is not perfect (PIM and PIM2 Hosmer-Lemeshow test χ^2^ 22 (*p* = 0.01) and χ^2^ 23 (*p* = 0.01), respectively) and the data are not spread evenly across the range of predicted risk (Fig. 1). However, the quantitative measures of performance suggest that the model can be considered ‘good’ (see Methods section).^21^ Cox's calibration regression showed an intercept −0.52 (95% CI, −0.85 to −0.19) and slope 1.19 (95% CI, 1.07 to 1.32) with χ^2^ 591 (*p* < 0.001) for PIM and, intercept −0.54 (95% CI, −0.86 to −0.22) and slope 1.19 (95% CI, 1.07 to 1.32) with χ^2^ 586 (*p*<0.001) for PIM2. The test of discrimination -area under the receiver operating characteristic curve - was 0.83 (95% CI, 0.80 to 0.86) for both models.

The Brier's score of overall fit was 0.05 in both models. The PIM2 model appears better in Fig. 1.

### Volume of PICU HI and neurosurgical practice

**Figure 2:**
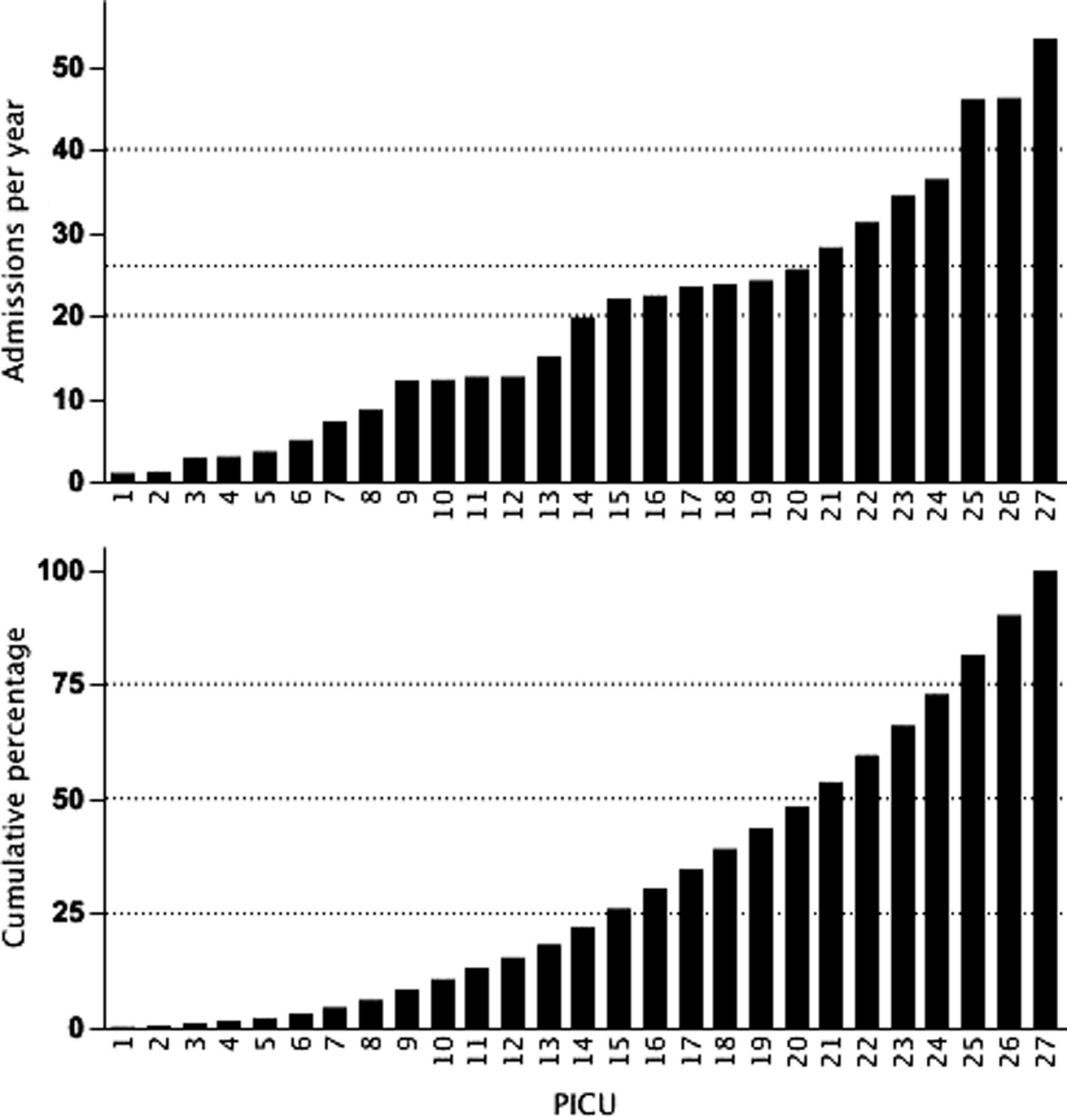
England and Wales PICU head injury practice by unit. *Upper panel:* Practice ordered according to size from smallest to largest. *Lower panel:* Cumulative practice by unit presented as percentage. The dotted lines in the lower panel show the borders of each quartile. The dotted lines in the upper panel show the size of practice defined at these quartile borders.

**Figure 3:**
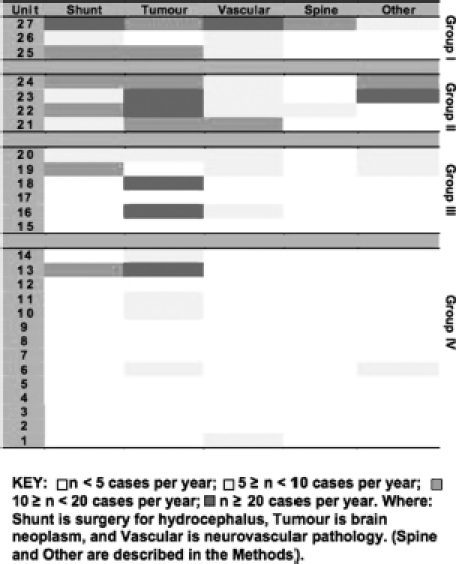
Specialist paediatric neurosurgical practice by PICU. Grouping of PICUs into quartiles defined in Fig. 2 from upperfirst to lowest-fourth, groups I to IV. PICU numbering is the same as in Fig. 2.

The system of PICU HI practice in England and Wales is illustrated in Fig. 2. The upper panel shows caseload for each PICU in ascending order of size (units 1 to 27). The lower panel shows cumulative practice in percentage, which forms a smooth curve with no obvious step-up or sigmoid shape. The borders between the four quartiles, from lower- to upper-quartile, coincide with units seeing more than 20, more than 25, and more than 40 HI admissions per year, respectively. Fig. 3 summarises specialist paediatric neurosurgical PICU practice by unit and grouped according to the divisions presented in Fig. 2: upper first quartile, group I (three PICUs); second quartile, group II (four PICUs); third quartile, group III (six PICUs); and, lower fourth quartile, group IV (14 PICUs). The numbering of the units is the same as in Fig. 2. It is evident that the majority of group IV units do not have significant volume of specialist paediatric neurosurgical practice admitted to the PICU. Group I units have the largest specialist paediatric neurosurgical PICU activity. The hierarchy in HI and paediatric neurosurgical PICU practice shown in Fig. 3 is also reflected in the volume of overall PICU practice per year in these groups of units (*p*<0.05). When all non-cardiac admissions are considered the median (IQR) admissions per year to PICUs in groups I to IV were 786 (618 to 1232), 399 (318 to 696), 493 (337 to 514) and 296 (198 to 377), respectively.

### PICU practice and mortality

During the management of head-injured children in the PICU, the more severe cases undergo MV and ICP monitoring; less severe cases are observed without any use of these interventions. In one-third (33.5%) of the episodes MV and ICP monitoring was used, in another half of the episodes (51.5%) MV alone was used, and in the remaining 15% observation without use of these interventions was undertaken. MV with ICP monitoring was used in older patients: median 10.9 (IQR 5.1-13.6) years *versus* 6.5 (2.2-12.1) years in those undergoing MV alone and 7.8 (2.9-12.6) years in those being observed without these interventions, *p*< 0.001.

In the risk-adjusted regression model there was no apparent effect of increasing individual PICU HI caseload on reducing mortality. There were significant effects for both intensive care intervention category (i.e. MV and ICP monitoring, MV alone, and observation without these interventions; *p*<0.001) and HI admissions by PICU grouping (i.e. group I-IV; *p*< 0.005) in the model. The odds ratio (OR) for death in the ICP monitoring category, in comparison to MV alone, was 2.08 (95% CI, 1.43 to 3.00;*p*< 0.0001). Table I summarises the grouped PICU data (i.e. groups I-IV) by category of interventions for HI practice (MV with ICP monitoring, MV alone, observation alone). There was some variation in case mix between the groups: group IV undertook fewer episodes of ICP monitoring and groups I and II had fewer episodes of observation without intervention. The MV with ICP monitoring category cases stayed longer in the PICU (median 6 days, χ^2^ 734, *p* < 0.0001) and a higher proportion received vasoactive drugs (70.9%, χ^2^ 1090, *p* < 0.0001). The SMR in the whole series of 2575 admissions was 1.06 (95% CI, 0.93 to 1.20), which indicates that the whole system performs as expected by the PIM model. On further inspection of Table I, a higher percentage of ICP monitoring patients died (13.2%) when compared with the percentage of death in the other categories (*p*<0.001). The SMR was greater than 1.00 in the ICP monitoring category (1.31, 95% CI 1.09 to 1.67; *p* = 0.004). This finding was due to group II and group IV data - SMR 1.58 (95% CI, 1.04 to 2.29; *p* = 0.02) and 1.83 (95% CI, 1.18 to 2.73; *p* = 0.004) respectively - since in the other two groups the lower limit of the 95% confidence interval for SMR was below 1.00. These statistics equate with up to 11 extra deaths in the 175 group II cases undergoing MV with ICP monitoring, or 14 extra deaths in the 117 group IV cases undergoing MV with ICP monitoring.

**Table I:** Summary of PICU group data by category of intensive care intervention for HI care

Group	ICP and MV	MV alone	non-MV	Summary
Group I				
*n* (%)	336 (46.2)	303 (41.6)	89 (12.2)^d^	728 (28.3)
Mortality (%)	48 (14.3)	26 (8.6)	2 (2.3)	76 (10.4)
SMR (95% CI)	1.22 (0.91-1.61)	0.81 (0.54-1.17)	-	1.00 (0.80-1.26)
LOS median (IQR)	6 (3-10) days	2 (1-4.5) days	2 (1-4.5) days	3 (2-7) days
Vasoactive drugs *n* (%)	232 (69.0)	53 (17.5)	1	286 (39.3)
Group II				
*n* (%)	175 (26.9)	397 (61.0)	79 (12.1)^d^	651 (25.3)
Mortality (%)	25 (14.3)	34 (8.6)	1 (1.1)	60 (9.2)
SMR (95% CI)	1.58 (1.04-2.29)^b^	1.04 (0.73-1.44)	-	1.16 (0.89-1.48)
LOS median (IQR)	7 (2-6) days	2 (1-2) days	2 (2-6) days	3 (2-6) days
Vasoactive drugs *n* (%)	116 (66.3)	54 (13.6)^f^	1	171 (26.3)
Group III				
*n* (%)	235 (36.9)	300 (47.1)	102 (16.0)	637 (24.7)
Mortality (%)	19 (8.1)	20 (6.7)	1 (1.0)	40 (6.3)
SMR (95% CI)	0.95 (0.59-1.47)	0.77 (0.48-1.16)	-	0.83 (0.60-1.13)
LOS median (IQR)	7 (4-12) days	3 (1-2) days	2 (2-7) days	3 (2-7) days
Vasoactive drugs *n* (%)	178 (75.7)	72 (24.0)	0	250 (39.2)
Group IV				
*n* (%)	117 (20.9)^a^	325 (58.2)	117 (20.9)	559 (21.7)
Mortality (%)	22 (18.8)	39 (12.0)	2 (1.4)	63 (11.3)
SMR (95% CI)	1.83 (1.18-2.73)^b^	1.10 (0.79-1.49)	-	1.27 (0.98-1.61)
LOS median (IQR)	6 (3-10) days	2 (1-2) days	2 (2-5) days	2 (2-5) days
Vasoactive drugs *n* (%)	86 (73.5)	61 (18.8)	2	149 (22.7)
All cases				
*n* (%)	863 (33.5)	1325 (51.4)	387 (15.1)	2575 (100)
Mortality (%)	114 (13.2)	119 (9.0)	6 (1.6)	239 (9.3)
SMR (95% CI)	1.31 (1.09-1.57)^c^	0.94 (0.79-1.13)	-	1.06 (0.93-1.20)
LOS median (IQR)	6 (4-11) days^e^	2 (2-4) days	2 (2-6) days	3 (2-6) days
Vasoactive drugs *n* (%)	612 (70.9)^e^	240 (18.1)	4	856 (33.2)

Groups I to IV as defined in Fig. 2. SMR, standardised mortality ratio; LOS, length of stay; ICP, intracranial pressure monitoring; MV, mechanical ventilation; non-MV, not mechanically ventilated.

Statistical tests:

a group IV significantly lower proportion in ICP and MV category;

b significantly higher and abnormal SMR in ICP and MV category in groups II and IV, *p*< 0.05;

c ICP and MV category significantly higher and abnormal SMR compared with the other categories, *p* = 0.004;

d significantly lower proportion of non-MV;

e where LOS and use of vasoactive drugs are significantly longer and higher in ICP and MV category;

f lowest frequency of use of vasoactive drugs in MV patients, in group II, *p*<0.05.

### Volume—outcome relationship

**Figure 4:**
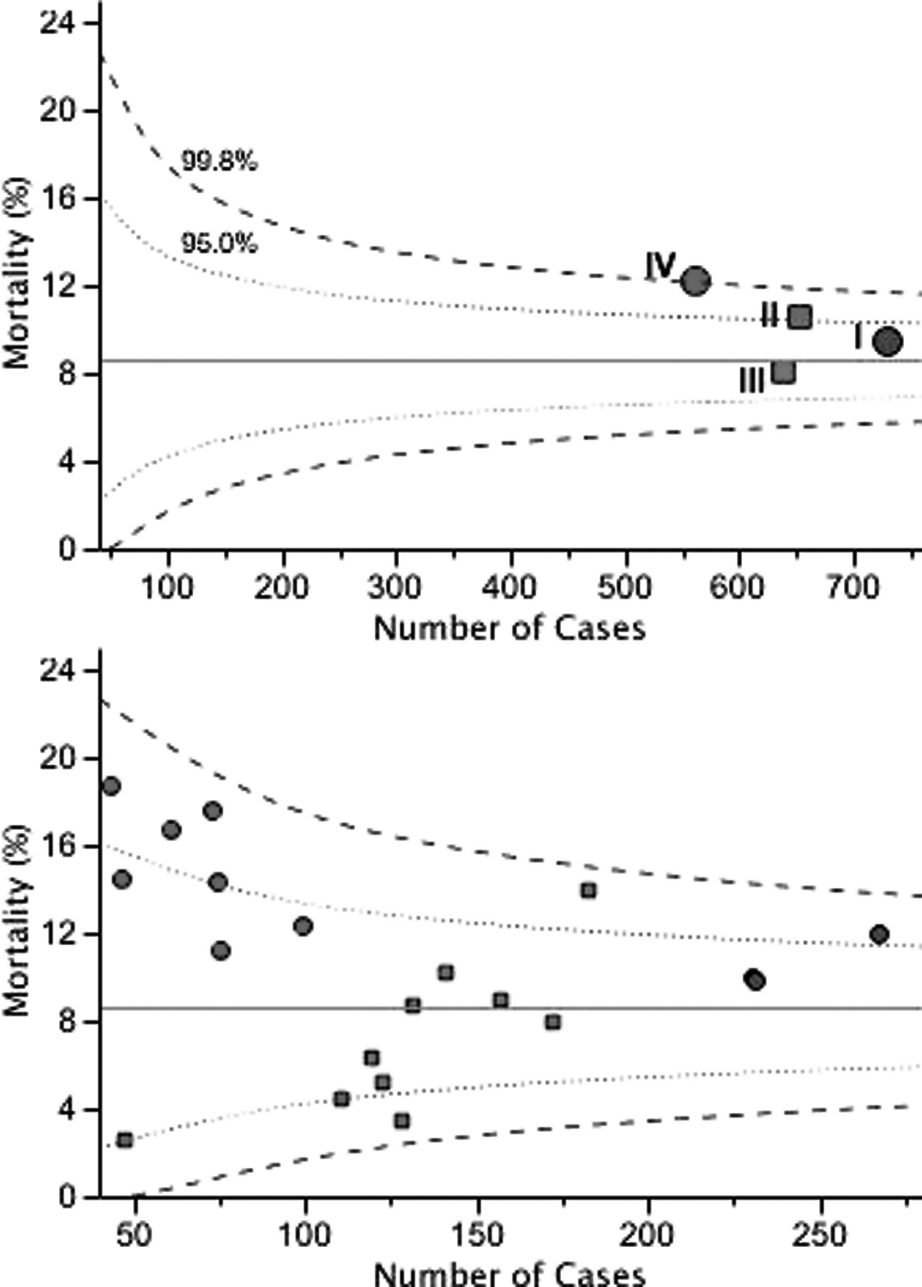
Funnel plots showing risk-adjusted mortality rate displayed as a scatter plot. The horizontal line shows the predicted mean 8.6%. Dotted lines show the 99.8% and 95% (2σ) limits. Points I–IV in the upper panel use the same notation as described in Fig. 3. Lower panel uses the same scheme of symbols, with each point representing PICUs within respective quartile-sectors of the health care system.

The relationship between volume and outcome is examined in the funnel plots in Fig. 4. The PIM2 risk-adjusted mortality in each quartile is displayed as a scatter plot and compared with the funnel plot (upper panel). Both PIM models have been examined in the analysis, but since PIM2 gives the more conservative results these are presented. In addition we examined the data for evidence of inter-unit transfer during the acute ictus and found only 8 instances where this occurred and none of these episodes end in death of the child. The figure shows that no sector lies outside the 99.8% limits. However, groups II (PICUs 21–24 in Fig. 2) and IV (PICUs 1–14 in Fig. 2) lie outside the 95% upper warning limit. Further scrutiny of individual PICUs within each quartile is displayed in the lower funnel plot. Each of the 20 PICUs with more than 40 cases in 2004-2008 is shown. No centre lies outside the 99.8% limits. Six of the PICUs had risk-adjusted mortality that was beyond the upper 2σ warning limit: 4 of 7 group IV PICUs and 1 unit in each of groups I and II. Half of the units in group III appeared to demonstrate evidence of possible best practice since their risk-adjusted mortalities were placed between the lower 95% and lower 99.8% limits.

**Figure 5:**
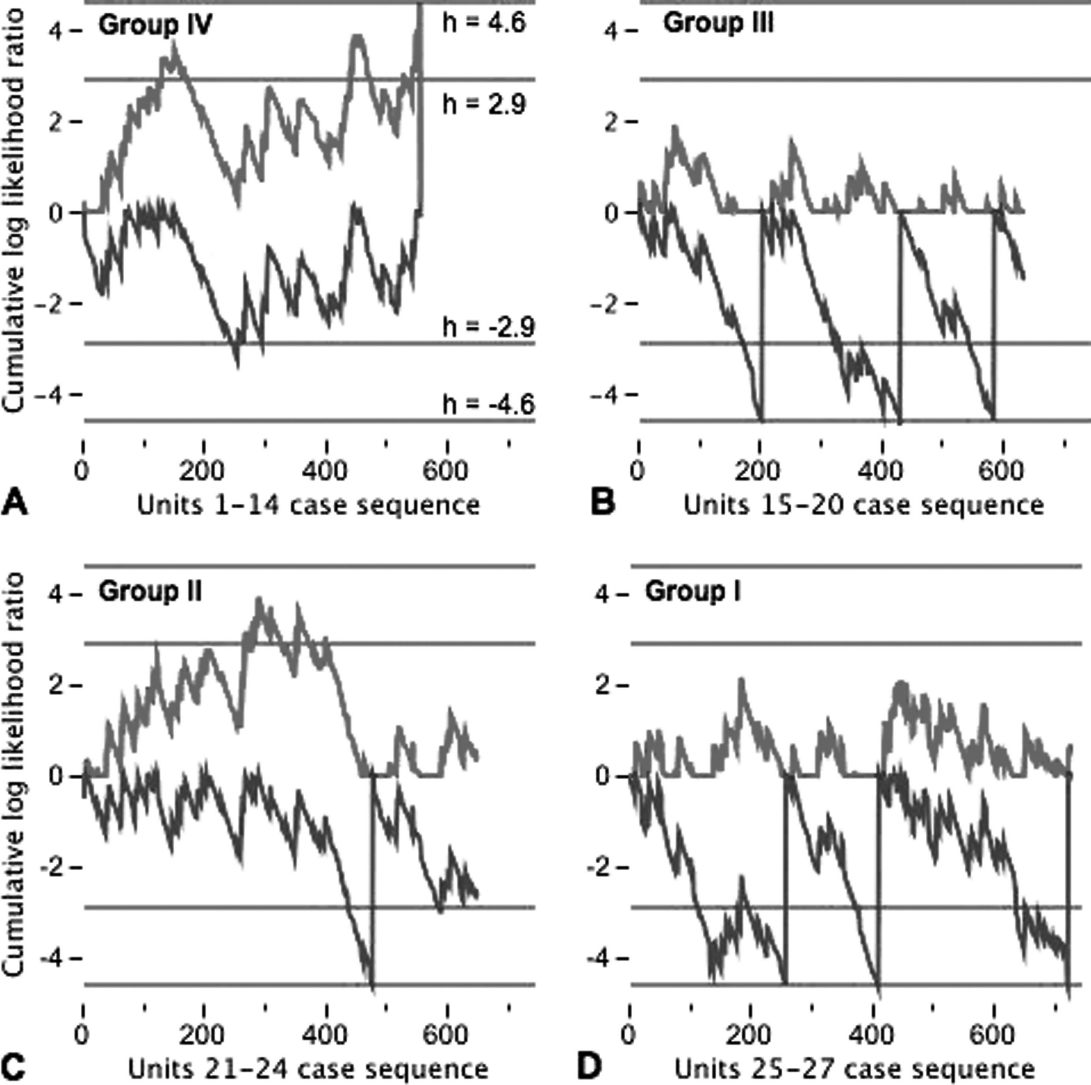
Half-cumulative-sum-risk-adjusted charts in the four quartiles of PICUs over their respective case series sequence, where: A, units 1–14, lower quartile and Group IV; B, units 15–20, third quartile and Group III; C, units 21–24, second quartile and Group II; D, units 25–25, upper quartile and Group I. Upper red line in each control chart tests for doubling of odds of death (*h*=4.6). Lower blue line in each control chart tests for halving of the odds of death (*h*= −4.6).

Table I and Fig. 4 together indicate there is excess mortality in groups II and IV, which may be a particular problem in those undergoing ICP monitoring. One difficulty with this analysis is that it considers experience during 2004-2008 as a whole. There is no insight into whether the finding represents earlier rather than more recent experience, which would be less relevant when considering contemporary performance in a system of care. The half-cumulative-sum-risk-adjusted charts shown in Fig. 5 displays performance with reference to PIM2 in the case series for each of the four groups. Fig. 5A is the control chart for group IV PICUs with plots for each episode in date and time sequence 2004-2008. Group IV PICUs have unexpectedly high number of deaths (upper red line in Fig. 5A crossing upper threshold after ∼550 cases). This finding is consistent with the observations in Table I and Fig. 4 and suggests that the finding is relevant to more contemporary practice. Fig. 5B (group III PICUs, average annual HI caseload 20–25) signals an unexpectedly low number of deaths after every 200 patients (lower blue line crossing lower threshold after 200, 400, and 600 cases). The trend is also continued after case 600. Better than expected performance is also signalled in Fig. 5C and D, but not with the same appearance in the plots. In Fig. 5C (group II PICUs, average annual HI caseload 20-35) there is only one signal for unexpected low number of deaths (after ∼500 cases) and the upper line crosses the 95% threshold between cases 300 and 400 in the sequence. In Fig. 5D (group I PICUs, average annual HI caseload 4 40) there are three signals for unexpectedly low number of deaths, but the cycle length for the most recent signal is ∼300 cases, which is greater than the cycle length in Fig. 5B.

## Discussion

In 2004-2008, in England and Wales, children needing intensive care after HI were managed in 27 different PICUs. Taken as a whole, performance of the system of care was as expected for risk of mortality, with no significant rate of excess death. Our analyses of PICU practice in HI care do not support the hypothesis that there is a simple relationship between PICU volume and performance. Rather, the hierarchy in increasing performance in the four volume sectors of the system was from lowest volume, to medium-higher volume, to highest volume, to medium-lower volume (see Fig. 4).

Before discussing the potential implications of these data for health care provision in England and Wales, we must discuss three possible limitations in our analyses. First, in this report, we have examined risk-adjusted mortality using the PIM/PIM2 model and the assumption in the interpretation of our analyses is that the instrument is valid. The model functions well in the paediatric HI population, but it is not perfect. If, however, one considers that the PIM model is undercalibrated (see Fig. 1), then our report of ‘performance as expected’ may actually mean poorer than expected performance. Such reasoning leads to the conclusion PICUs in groups II and IV are, in fact, underperforming (see control limits in Fig. 4).

The second possible limitation of the study is the lack of GCS score in the PIM model. Rather, the model uses derangement in cardiorespiratory parameters to quantify severity and the relevance of these to assessment of mortality risk in HI may be questioned. However, it is well recognised in severe cases of HI in adults and children that hypoxia and hypotension are significant predictors of outcome.^27^-^30^ The problem with the GCS score is that with modern resuscitation practices it is unreliable as a guide to severity for epidemiological purposes because more severe cases undergo emergency endotracheal intubation and supportive MV.^31^,^32^ In adults, lowest GCS score from the first 24 hours in the intensive care unit has good discrimination in a risk prediction model in adult HI admissions, but only in those who are not sedated or paralysed for the entire first 24 hours.^21^ Also, in adults with severe HI, all other intensive care risk prediction models out perform the GCS score.^21^ Taken together, we consider the PIM model to be valid in the paediatric HI population.

The third possible limitation of this study is the size of the population. Critical care for child HI is small in regard to the broad picture of PICU practice, accounting for only 4.4% of admissions for non-cardiac surgical intensive care in England and Wales. There is no getting around this problem and we believe that the 5-year dataset is probably the limit of what is reasonable for collection of an accumulated population without there being significant influences from changes in practice or health system reorganisation. One consequence of a small population (∼2500) managed in 27 different PICUs is that only 4 units saw 20 or more deaths, thereby invalidating the use of an individual SMR statistic in each of the other 23 units. Hence we needed to resort to studying pooled cohorts (or quartiles) in the system 22, 24

In the field of paediatric neurosurgery and critical care^33^,^34^ it is important to know whether volume of PICU HI practice has an impact on mortality. We have explored this relationship along with information about volume of specialist paediatric neurosurgical activity on the PICU and conclude that the health care system as a whole performs as expected. A new question is whether the health care system for critically ill head-injured children can be improved. Further work is needed, particularly as we are not able to explore the reasons why some head injured patients were managed in low-volume PICUs with little or no specialist paediatric neuro-surgical activity (group IV). Given the varied geographical regions found in the UK - ranging from metropolitan zones to more dispersed suburban and rural areas - there may be practical reasons why patients were not transferred to larger centres. Cases may have even been managed remotely in consultation with the paediatric neuro-surgical centre. That said, we believe the PICUs that are involved in this apparent poor-performing sector of the health care system should examine and discuss their operational procedure for severe HI in children.

Last, it is not clear what accounts for the hierarchy in performance we have observed in child HI PICU care. Co-location with specialist paediatric neuro-surgical PICU activity is not a guarantee for success. However, the sector with the poorest performance, comprising 14 PICUs, undertook little or no specialist paediatric neurosurgical activity on the unit. Our analysis also indicates that the major difference in outcomes is in those undergoing ICP monitoring. There was no difference in the surrogate markers of this practice such as use of inotropes or duration of PICU stay. However, we have not been able to fully address the detail and aggressiveness of therapies in individual cases in this analysis. Neither have we been able to address more fundamental system factors such as co-location with an adult neurotrauma centre, the nature of neurosurgical involvement and supervision in PICU HI care, staffing levels, or standardised medical management.^9^ Each of these factors should be explored in further studies.

## List of NHS Trusts and hospitals participating in PICANet 2004–2008

Addenbrooke's NHS Trust: Addenbrooke's Hospital. Bart's and the London NHS Trust: Bart's and The Royal London. Birmingham Children's Hospital NHS Trust: Diana, Princess of Wales Children's Hospital. Brighton & Sussex University Hospitals NHS Trust: Royal Alexandra Hospital for Sick Children. Cardiff & Vale NHS Trust: University Hospital of Wales. Central Manchester & Manchester Children's University Hospitals NHS Trust: Royal Manchester Children's Hospital. Great Ormond Street Hospital for Children NHS Trust: Great Ormond Street Hospital for Sick Children. Guy's & St. Thomas' Hospital NHS Trust: Guy's Hospital. Hull & East Yorkshire Hospitals NHS Trust: Hull Royal Infirmary. King's College Hospital NHS Trust: King's College Hospital. Leeds Teaching Hospitals NHS Trust: Leeds General Infirmary, St. James University Hospital. Newcastle upon Tyne Hospitals NHS Trust: Newcastle General Hospital, Freeman Hospital, Royal Victoria Infirmary. Oxford Radcliffe Hospitals NHS Trust: John Radcliffe Hospital. Queen's Medical Centre Nottingham University NHS Trust: Queen's Medical Centre. Royal Brompton & Harefield NHS Trust: Royal Brompton Hospital. Royal Liverpool Children's NHS Trust: Alder Hey Hospital. Royal Group of Hospitals and Dental Hospital HSS Trust, Belfast Hospital for Sick Children Sheffield Children's NHS Trust: Sheffield Children's Hospital. Southampton University Hospitals NHS: Trust Southampton General Hospital. South Tees Hospitals NHS Trust: James Cook University Hospital. St. George's Healthcare NHS Trust: St. George's Hospital. St. Mary's NHS Trust: St. Mary's Hospital. The Lewisham Hospitals NHS Trust: University Hospital, Lewisham. University Hospitals Bristol NHS Foundation Trust: Bristol Royal Hospital for Children. University Hospitals of Leicester NHS Trust: Leicester Glenfield Hospital, Leicester Royal Infirmary. University Hospital of North Staffordshire NHS Trust: Stoke on Trent City General.

## PICANet Steering Group members

Mrs Pamela Barnes, Professor Nick Black (Chair) (2002–2007), Mr William Booth, Ms Bev Botting Child (2002–2003), Dr. Jean Chapple (2002–2006), Dr. Bill Chaudhry (2002–2003), Dr. Anthony Chisakuta, Dr. Mark Darowski, Mr Noel Durkin, Dr. Ian Jenkins, Dr. Steve Kerr, Ms Helen Laing (2004–2006) & (present), Mr Ian Langfield (2002–2003), Lucy Lloyd Scott, Dr. Michael Marsh, Dr. Jillian McFadzean, Dr. Roddy McFaul (2002–2003), Dr. Kevin Morris, Professor Jon Nicholl (2002–2006), Dr. Roddy O'Donnell, Dr. Gale Pearson, Dr. Mark Peters, Ms Tanya Ralph (2002–2006), Ms Laura Reekie, Dr. Kathy Rowan, Mr Stuart Rowe, Ms Dominique Sammut, Dr. Jenifer Smith, Dr. Charles Stack (2002–2006), Professor Stuart Tanner (2003–2006), Dr. Robert Tasker (2004–2008), Dr. Edward Wozniak.

## PICANet Clinical Advisory Group members

Dr. Paul Baines (2002–2008), Ms Corenna Bowers (2002–2004), Faith Chinanga, Kathryn Claydon- Smith, Dr. Gillian Colville, Dr. Anthony Chisakuta, Dr. Peter Davis, Dr. Andrew Durward, Ms Georgina Gymer (2005–2006), Dr. James Fraser (2002–2005), Dr. Hilary Klonin, Ms Helen Laing, Ms Christine Mackerness, Tina McClelland, Dr. Jillian McFadzean, Ms Victoria McLaughlin, (2002–2007), Elizabeth McKinty, Dr. Roddy O'Donnell, Ms Geralyn Oldham, Dr. Gale Pearson (Chair), Dr. Damian Pryor (2002–2004), Chloe Rishton, Dr. Allan Wardhaugh, Ms Debbie White.
